# A Lightweight Secure Adaptive Approach for Internet-of-Medical-Things Healthcare Applications in Edge-Cloud-Based Networks

**DOI:** 10.3390/s22062379

**Published:** 2022-03-19

**Authors:** Abdullah Lakhan, Ali Hassan Sodhro, Arnab Majumdar, Pattaraporn Khuwuthyakorn, Orawit Thinnukool

**Affiliations:** 1 Department of Computer Science, Dawood University of Engineering and Technology, Karachi 74800, Pakistan; abdullah.lakhan@duet.edu.pk; 2 College of Computer Science and Artificial Intelligence, Wenzhou University, Wenzhou 325035, China; 3 Department of Computer Science, Kristianstad University, SE-291 88 Kristianstad, Sweden; ali.hassan_sodhro@hkr.se; 4 Department of Civil and Environmental Engineering, Imperial College London, London SW7 2AZ, UK; a.majumdar@imperial.ac.uk; 5 College of Arts, Media, and Technology, Chiang Mai University, Chiang Mai 50200, Thailand; pattaraporn.khuwuth@cmu.ac.th

**Keywords:** neighborhood search, secure offloading, dynamic approaches, workflow healthcare applications, LSEOS, healthcare, scheduling

## Abstract

Mobile-cloud-based healthcare applications are increasingly growing in practice. For instance, healthcare, transport, and shopping applications are designed on the basis of the mobile cloud. For executing mobile-cloud applications, offloading and scheduling are fundamental mechanisms. However, mobile healthcare workflow applications with these methods are widely ignored, demanding applications in various aspects for healthcare monitoring, live healthcare service, and biomedical firms. However, these offloading and scheduling schemes do not consider the workflow applications’ execution in their models. This paper develops a lightweight secure efficient offloading scheduling (LSEOS) metaheuristic model. LSEOS consists of light weight, and secure offloading and scheduling methods whose execution offloading delay is less than that of existing methods. The objective of LSEOS is to run workflow applications on other nodes and minimize the delay and security risk in the system. The metaheuristic LSEOS consists of the following components: adaptive deadlines, sorting, and scheduling with neighborhood search schemes. Compared to current strategies for delay and security validation in a model, computational results revealed that the LSEOS outperformed all available offloading and scheduling methods for process applications by 10% security ratio and by 29% regarding delays.

## 1. Introduction

The development of digital healthcare using the Internet of Medical Things (IoMT) expands day by day as the number of technologies in practice increases [1]. The IoMT is an architectural infrastructure consisting of user, edge, and cloud layers. The user layer consists of sensor devices connected to devices such as mobile devices and smart watches. The edge layer is cloud resources implemented at the edge network, with less communication delay. The cloud layer consists of rich resources and exists multiple hops away from user devices. Many healthcare applications are designed on the basis of service-oriented architecture (SoA) and run in these three layers. These medical applications connect to various biosensors, and send their information to cloud and edge servers for additional evaluation. Heartbeat ECG, EEG, blood pressure, and oxygen levels in the human body, for example, can be monitored by biosensors, and application information is sent to nearby hospital servers for analysis [2]. To fulfil the healthcare monitoring purpose in the system, the IoT network incorporates diverse elements such as healthcare sensors, wearable networks, and mobile cloud services [3]. The IoT network offloads and shares data of healthcare applications to the different layers, such as network and computing layers, for processing based on their given constraints. However, there are many risks due to other layers in the network [4]. Natural hazards are data security, delays, workload failure due to attacks, and the unavailability of data in the system. Therefore, security-enabled data offloading and processing are important challenges in IoT networks for healthcare applications [5].

Many IoT network-enabled algorithms were suggested for healthcare applications to protect data and store them cryptographically [6]. These static heuristics, such as heterogeneous earliest finish time, genetic algorithm, simulated annealing, and particle swarm optimization, are widely implemented to run healthcare applications in polynomial time [7]. Local and global searches (e.g., simulated annealing and tabu search) are fundamental guided candidate solutions to the aforementioned algorithms to achieve optimal results of healthcare applications in heterogeneous fog cloud networks [8]. There are two types of edge cloud networks widely implemented in the literature, namely, homogeneous and heterogeneous nodes. On the basis of nodes, different studies suggested static and dynamic approaches based on heuristic and guided random search for combinatorial convex optimization research problems of healthcare applications in both heterogeneous and homogeneous environments. Security algorithms implemented inside these heuristics include SHA-256, MD5, CRC32 based on AES and RSA keys in the heterogeneous fog cloud nodes for healthcare applications [9,10,11,12].

However, these algorithms present many research challenges when they are implemented inside distributed heterogeneous fog cloud networks for delay-optimal and adaptive healthcare applications in the network. (i) Existing scheduling approaches only focused on resource availability, energy, and security mechanisms in healthcare applications’ heterogeneous fog cloud nodes. Static and dynamic scalability can be managed with these methods. However, these methods consume much more resources and energy when they integrate the security mechanism into an IoT network for healthcare applications. (ii) All existing IoT networks have approaches to execute datasets, including healthcare workloads, and they widely ignore workflow healthcare applications in a heterogeneous network. Therefore, IoT network approaches for workflow applications need to be designed in heterogeneous and homogeneous environments for execution. (iii) All existing security approaches are static and consume much more resources in a cryptoprocess on offloaded data from applications. Therefore, a lightweight and adaptive security approach must be designed for healthcare applications in the network.

This study develops the LSEOS metaheuristic, which contains several phases: adaptive sequencing and internal deadlines, adaptive two-way security validation, adaptive task scheduling, and neighborhood search. The goal of the research was to reduce application delays, such as mobile, edge, and cloud execution times. The study examines workflow applications for a variety of jobs, including mobile, edge, and cloud workloads. At the design and development of apps, the tasks are annotated. This research takes into account heterogeneous computing nodes, namely, mobile, edge, and cloud computing.

The manuscript is organized as follows. Section 2 discusses existing IoT network efforts for healthcare applications in homogeneous and heterogeneous fog cloud environments. Section 3 describes the study’s proposed architecture and problem formulation. Section 4 shows the flow of proposed heuristics and their steps for the problem solution. Section 5 shows how the proposed work was implemented, and optimal results were obtained compared to existing studies. Section 6 shows the contribution and achievements of the proposed lightweight scheme, and a future road map of the current research.

## 2. Related Work

This part discusses the literature approaches (e.g., static and dynamic) of IoT networks for healthcare applications. Fundamental methods are offloading and scheduling to monitor and schedule healthcare tasks in a system. Metrics and constraints of existing studies are represented in Table 1.

In [1,2], the authors investigated the task offloading and scheduling problem on the basis of a polynomial. These studies suggested genetic-algorithm-based solutions with a secure hashing algorithm (256 bits) for healthcare applications in IoT technologies. These studies obtained different objectives such as response time, tardiness, and network delay. An integrated particle swarm optimization-enabled scheme was presented by [3,4,15,17], where a message digest (MD5) scheme was integrated with a particle swarm optimization (PSO) algorithm to secure healthcare tasks in the IoT network. The goal was to minimize security risks in the network, and these studies considered fine-grained tasks and homogeneous networks in their models. The authors in [5,6] suggested a static improved algorithm based on an ant-colony metaheuristic, and integrated a cryptography algorithm to protect and share secure data between nodes. The considered computing nodes were homogeneous in the IoT network, where these nodes are placed at the edge of the user network.

In [7,8,9,10,11,12,13,14,15,16], the authors suggested local and global searching (simulated annealing and genetic algorithm)-enabled dynamic approaches to solve the offloading and and scheduling problem in IoT networks. The main goal was to reduce local and global search times for scheduling on heterogeneous fog and cloud nodes in the system, establish a secure environment among connected nodes, and minimize attack risk in the IoT network. These dynamic approaches can identify anomalies and resource performance of applications, but there was still uncertainly of heterogeneous nodes in terms of scalability [17,18,19,20].

Machine-learning-enabled convolutional neural network (CNN), k-nearest neighbors (KNN) and support vector machines (SVM) are machine-learning algorithms that support security in adaptive and learning ways. On the basis of these methods, a security mechanism was introduced in IoT networks for the healthcare applications in different works. These studies implemented closely related existing schemes in the simulation, for instance, delay optimal long short-term memory (LSTM) [21], workflow metaheuristic system (WFMS), [22] and workflow metaheuristic cloud (WMC) [23]. These studies are closely related to our work to execute workflow applications on heterogeneous nodes in cloud computing. In [24,25,26,27,28,29,30], the authors suggested dynamic approaches to deal with runtime anomalies and security risks in IoT networks. However, these methods consumed too many resources and had much delay during encryption and decryption in resource allocation.

Many studies [31,32,33,34,35] mobility-enabled fog and edge cloud networks devised for healthcare applications to offer mobility-aware services to mobile patients. The goal was to minimize location offloading risk and unavailability of services in the network. In these works, models offered online remote services from different locations and supported mobility-aware services to patients.

To the best of our knowledge, lightweight secure offloading and scheduling for workflow applications has not been studied yet. The novelty of secure offloading is that the proposed method is lightweight, and consumes fewer resources and time to process security mechanisms and offloading in a mobile edge cloud network. Existing security offloading techniques consume much more resources and time when processing IoT healthcare applications in a network. Therefore, the dynamic and secure offloading scheme is lightweight and robust, and meets the requirements of IoT healthcare applications in the network. Generally, dynamic scheduling methods are those in which tasks are prioritized at runtime in the system.

## 3. Proposed LSEOS Metaheuristic and Architecture

**Figure 1:**
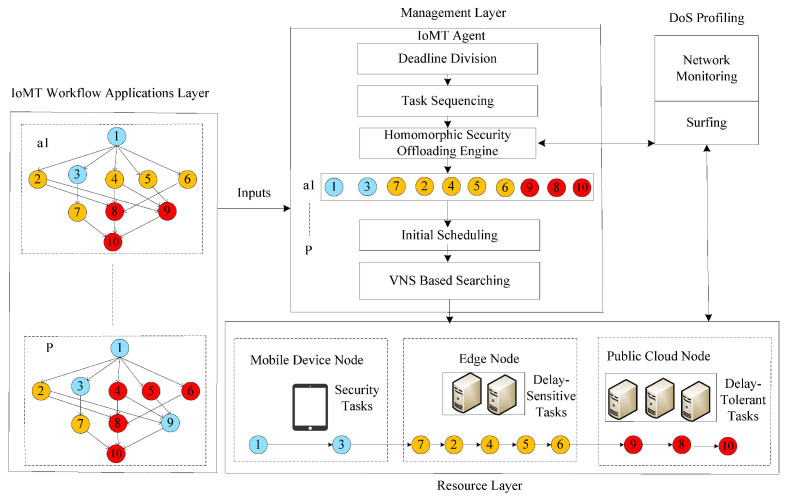
LSEOS metaheuristic and architecture.

As illustrated in Figure 1, a lightweight and delayed optimal secure Internet of Medical Things (IoMT) network was devised for workflow healthcare applications. The goal was to provide optimal offloading and scheduling in a network that are both secure and delay-optimal. The suggested architecture comprises layers for IoT (i.e., IoMT) workflow applications, management, and resources. At the design time of applications, the program was the workflow and consisted of three processor tasks in the system, namely, local, fog, and cloud tasks. Local tasks must be completed on mobile devices with the least delay and amount of time while sharing data with the system’s edge node tasks.

Edge tasks are simultaneously scheduled at available edge nodes for further processing and data collection from local tasks before being executed. The cloud tasks merely store the application’s data-intensive results in the cloud without compromising the system’s application performance. The IoMT network’s management layer comprises various dynamic offloading and scheduling methods. The IoMT agent, which is more adaptive and handles all application operations at any time in the system, was devised in this study. The IoMT agent is the primary handler and combines several approaches: deadline division, topological order sequencing, a fully homomorphic mechanism, preliminary task assignment (initial scheduling), and variable neighborhood adaptive resource seeking for tasks. Denial of service (DoS)-aware techniques were developed on the basis of network surfing and system monitoring without compromising the system’s application performance. The problem’s symbolic notations and descriptions are defined in Table 2.

### 3.1. Problem Formulation and System Model

**Figure 2:**
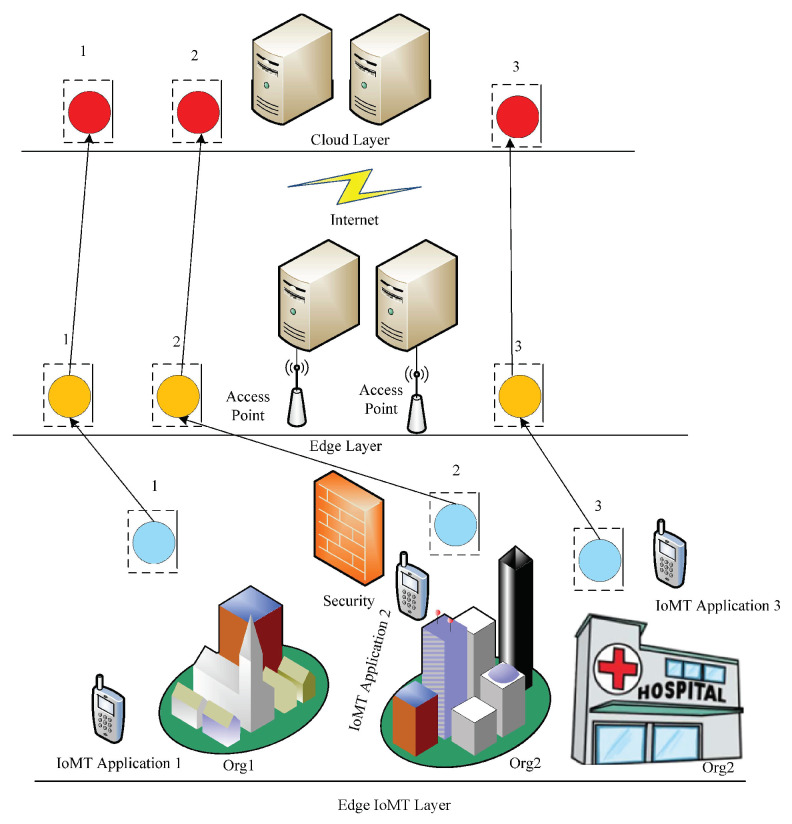
IoMT system model.

The problem was formulated as follows. The execution scenario of workflow applications in an IoMT network was first discussed as shown in Figure 2.

Real practical scenarios of healthcare hospitals as different organizations (Org) were considered. Many healthcare sensors were connected with varying healthcare organizations, and their data were offloaded for processing via access points at edge computing. Local tasks were executed in the IoT (mobile devices), and edge tasks were offloaded to the edge computing layer for execution. At the same time, delay-tolerant tasks were offloaded to the cloud via the Internet, as shown in Figure 2.

The mathematical formulation of the study was as follows. *P* number of workflow applications were represented by a directed acyclic graph. The workflow application had starting tasks *i* and *j*, and there was communication between them. Each workflow application had workload $w_{a}$ and deadline $deadline_{a}$.

Three different computing nodes were considered: mobile device *m*, edge server *e*, and cloud computing *c*. Each node had a different processing speed $\zeta_{m}$, $\zeta_{e}$, $\zeta_{c}$, and resources $\epsilon_{m}$, $\epsilon_{e}$, $\epsilon_{c}$ in the system.

### 3.2. Task Annotation at Design Time

The application divides the workload into three task types: mobile, edge, and cloud tasks. Mobile tasks must be locally executed on mobile devices. These tasks are locally encrypted and decrypted with the proposed secure algorithm. Then, encrypted data are offloaded to edge tasks for execution. After executing tasks, the data are offloaded to cloud computing to complete cloud tasks in the system.

### 3.3. Workflow Application Characterization

**Figure 3:**
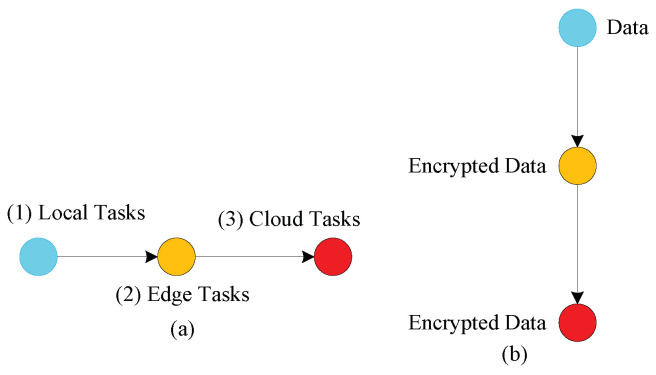
Workflow applications on distributed mobile ege cloud networks in Proposed Architecture.

The considered application contained three different task types: mobile (represented by blue nodes), edge (yellow nodes), and cloud (red nodes) tasks, as shown in Figure 3.

Figure 3a denotes the sequence execution of tasks from local to cloud tasks with different requirements. Due to data security, local tasks encrypt and decrypt locally at the local machine, and then offload their cipher data to edge tasks for further execution. Cipher data are never interpreted at edge nodes, and computation is applied on the cipher data to complete their process. Edge nodes, on the other hand, send their executed cipher data to the cloud node for further storage as shown in Figure 3b.

### 3.4. Mobile Computing Assignment

In this section, we determine the execution time of mi tasks at local devices, which locally had encryption and decryption time at mobile devices. Local execution time is determined for local mobile tasks in the following way.

(1) $$M_{e}^{mi}=\sum_{mi=1}^{a}\frac{Enc}{\zeta_{m}}+\frac{Dec}{\zeta_{m}}\times x_{mi=1}$$

Equation (1) determines the encryption and decryption time of local tasks at mobile device. For homomorphic encryption, cryptographic modeling was suggested on the basis of the El Gamal scheme. The homomorphic mechanism consists of three main elements: (1) key generation, (2) encryption time, and (3) a decryption-time-based asymmetric public key. Encryption and decryption based on an asymmetric public key are locally determined in the following way.

(2) $$\begin{array}{c} Enc=Pk\leftarrow data_{i}\leftarrow SHA-256. \end{array}$$

Equation (2) determines the encryption time (PK(public key) in the system.

(3) $$\begin{array}{c} Dec=PV\leftarrow Enc\leftarrow data_{i}. \end{array}$$

Equation (3) determines the decryption time with the private key (PV) in the system.

### 3.5. Edge Computing Assignment

Data are offloaded from the mobile device to the edge node in the form of cipher text. Therefore, the execution time on the edge nodes consists of communication and processing times between mobile devices, and the edge is determined in the following way.

(4) $$E_{e}^{ei}=\sum_{ei=1}^{a}\frac{Enc}{\zeta_{e}}+\frac{data_{i}\leftarrow Enc}{bw_{upload}}↔\frac{data_{i}\leftarrow Enc}{bw_{download}}\times x_{ei=2}.$$

Equation (4) determines the execution time of encrypted tasks, and the communication between mobile device and edge with both uploading and downloading data.

### 3.6. Cloud Computing Assignment

Cloud computing only stores generated data from the edge nodes. Therefore, execution on encrypted executed data for uploading and downloading is determined in the following way.

(5) $$C_{e}^{ci}=\sum_{ci=1}^{a}\frac{Enc}{\zeta_{c}}+\frac{data_{i}\leftarrow Enc}{bw_{upload}}↔\frac{data_{i}\leftarrow Enc}{bw_{download}}\times x_{ci=3}$$

Equation (5) determines the execution time of encrypted tasks, and communication between cloud and edge with both uploading and downloading data. The total execution and communication times of all tasks on all nodes are determined in the following way.

(6) $$Delay=\sum_{a=1}^{P}\sum_{mi=1}^{a}\sum_{ei=1}^{A}\sum_{ci=1}^{A}M_{e}^{mi}+E_{e}^{ei}+C_{e}^{ci},\;\forall a=1,\ldots ,P.$$

Equation (6) calculates the total delay time of workflow application a at different computing nodes. The problem formulation based on linear programming was designed in the following way.

(7) minDelay,a=1,…,P.

Equation (7) is the combinatorial convex objective of the considered problem in the system.

(8) $$\sum_{a=1}^{mi,ei,ci}data_{i}\leq \epsilon_{m,e,c}.$$

Equation (8) ensures that, before execution on the application, there are enough resources in all computing nodes for the processing.

(9) $$\sum_{a=1}^{P}data_{i}\leq deadline_{a},\;a=1,\ldots ,P.$$

All applications must be executed before their deadlines as defined in Equation (9).

## 4. Proposed Security Efficient Offloading and Task-Scheduling (LSEOS) Metaheuristic Approach

The combinatorial convex optimization problem for heterogeneous tasks and parallel computing nodes is always challenging. This study also considers different workflow tasks such as mobile, delay-sensitive, and delay-tolerant, and heterogeneous computing nodes such as mobile devices, edge nodes, and cloud computing in the system. The goal was to execute workflow applications on different nodes in order to minimize the total delay of applications. Existing offloading schemes [1,4,7] only focused on coarse- and fine-grained applications. Therefore, there are no particular architectures and schemes for workflow applications. This study devised a lightweight security-enabled efficient offloading and scheduling (LSEOS) algorithm framework that consists of different schemes to minimize the delay of applications in the architecture. The proposed SEOS, as shown in Algorithm 1, comprises different schemes, and was determined as follows.
**Algorithm 1:** LSEOS metaheuristic.
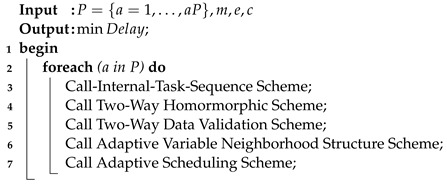



**Figure 4:**
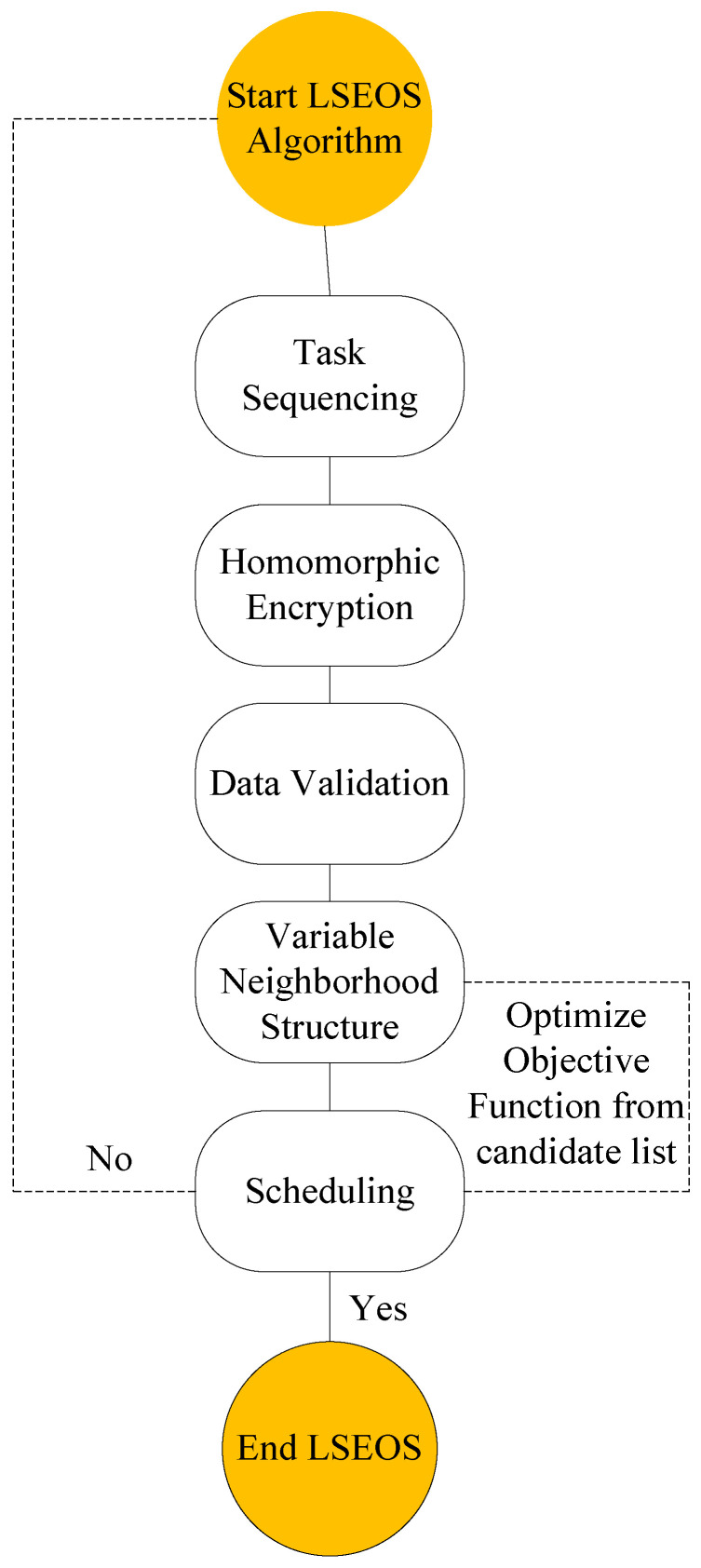
LSEOS metaheuristic algorithm for the considered problem.

Figure 4 shows the LSEOS metaheuristic process from the initial to the end component for the considered problem. It starts from input and ordering all tasks on the basis of their quality-of-service (QoS) requirements in the system. The primary goal of the sequence is to order all tasks on the basis of their priorities such as deadlines and delays in the system. Once the tasks are sorted, there is two-way homomorphic encryption. This encryption mechanism encrypts and decrypts data at local devices. Fog and cloud nodes process these tasks on the basis of their ciphtertext instead of plaintext in the system. Two-way validation ensures the validation of data between nodes. The candidate solution is the searching mechanism inside LSEOS that finds random optimal solutions and replaces the existing scheduling solution when its status is “Yes”. If tasks are failed, it generates a “No” status and reprocesses all tasks from start in the system, as shown in Figure 4.

### 4.1. Adaptive Task Sequencing Rule Scheme

There are many sorting techniques that are widely exploited in the workflow applications, for instance, deadline-enabled sorting, earliest deadline finish time, earliest finish time, and smallest workload first. However, these techniques only sort tasks on a single group of same applications. However, workflow application tasks are divided into three sets: local, delay-sensitive, and delay-tolerant tasks. Therefore, these methods cannot be directly applied on different sets of tasks, and internal adaptive task sequencing rules are suggested in which all tasks are sorted according to sets.

Algorithm 2 initially assigns a deadline to tasks at different levels, such as mobile, delay-sensitive, and cloud tasks, on the basis of their execution time. All tasks are sorted on the basis of their deadlines. Assigned deadline and sorting are adaptive rules that assign the deadline and sorting to workflow applications at the runtime of submission in the system. Algorithm 2 determines the deadline and sequencing rules in the following way. Deadline: a={i=1←30s,i=3←40s,i=2←30s,i=4←50s,i=5←60s,i=6←30s,i=9,i=8,i=10←89s}. After deadline assignment, tasks are sorted as follows: a={i=1,i=3,i=2,i=4,i=5,i=6,i=9,i=8,i=10}∀a=1,…,A.
**Algorithm 2:** Adaptive task sequencing rule scheme.
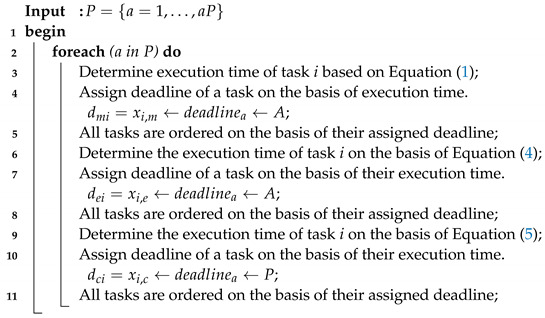



### 4.2. Two-Way Secure Offloading Scheme

Algorithm 3 determines the two-way homomorphic security mechanisms where denials of service are monitored via network profiler scheme before offloading any data from any node. Surfing is a mechanism that generates the report of intrusion nodes in the system. Algorithm 3 determines the stability of nodes before offloading any data to any node. Data for the encryption are computed on the basis of a 256-bit public key, and two random long integers always generate random keys on given task data in the algorithm. The network profiler always checks the status of security in the algorithm. The data can be decrypted on the basis of a private key at the mobile devices, and results are accessed from the cloud node.
**Algorithm 3:** Lightweight two-way homomorphic security scheme.
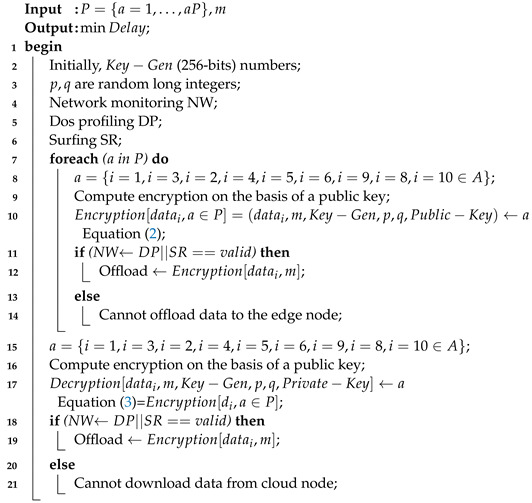



### 4.3. Adaptive Task Scheduling and Neighborhood Structure

The two ways of offloading are from mobile devices to edge node and from edge node to cloud computing. Adaptive task scheduling is a mechanism where execution time is divided among mobile devices, edge nodes, and cloud computing. There are many existing algorithms that can schedule different nodes in the literature, for instance, heterogeneous earliest finish time algorithm, genetic algorithm, PSO, and ant colony. With these algorithms, heterogeneous jobs can be run on heterogeneous cloud nodes. However, these algorithms cannot work with encryption at the edge and cloud nodes to process workflow applications. Workflow applications have different requirements, for instance, encryption can be performed on one node, and other nodes must compute the encrypted data and not the plaintext. A novel task-scheduling and variable neighbour-searching scheme, Algorithm 4, was devised.
**Algorithm 4:** Adaptive task-scheduling and variable neighbor-searching scheme.
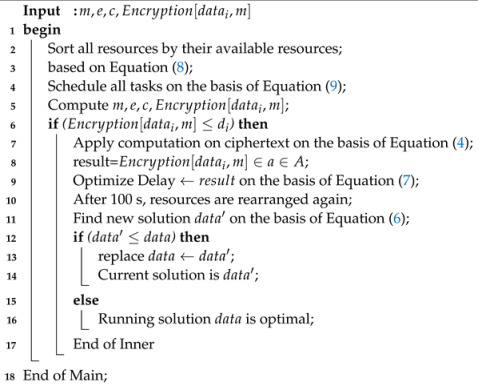



## 5. Performance Evaluation

In this section, existing performance workflow schemes [15,17,19,20] are compared with proposed scheme LSEOS on the basis of the performance of workflow applications. The IFogsim framework was used, where proposed and existing approaches were efficiently implemented and their performance in the architecture was evaluated. Table 3 shows the parameter settings of the proposed architecture, which was implemented on the basis of IFogsim for the experiments.

### 5.1. Parameter Settings of Simulation Environment of the Considered Problem

In this part, the study showed the simulations parameters of the considered problem and showed how to conduct the experiments based on the proposed and baseline approaches.

To evaluate the performance of IoMT workflow tasks, we designed different flows of different types of workflow tasks, namely, a set of security, delay-sensitive, and delay-tolerant tasks at design time. Security must be locally executed with private encrypted and decrypted keys, delay-sensitive tasks are offloaded to the edge node for execution, and delay-tolerant tasks should be executed onto the remote cloud. All types are represented by different nodes. Blue nodes are security tasks, light yellow nodes are delay-sensitive tasks, red circles show delay-tolerant tasks, and other types were randomly designed.

Table 4 denotes the workflow applications and their tasks. Each workflow application consisted of three types of tasks, as shown in Table 4: mobile, edge, and cloud tasks; they are processed at different nodes in the system. All tasks were part of a workflow, some had original data, and some shared their data for processing. All tasks were constrained by their predecessor and successor in the system.

The objective function of the study, that is, delay measured in terms of microseconds (ms) for workload assignment to the distributed nodes. We now compare the results of IoMT workflow tasks with the proposed framework, its components, and existing offloading and scheduling frameworks. Component results are discussed below.

### 5.2. Delay Optimal Result Comparison

**Figure 5:**
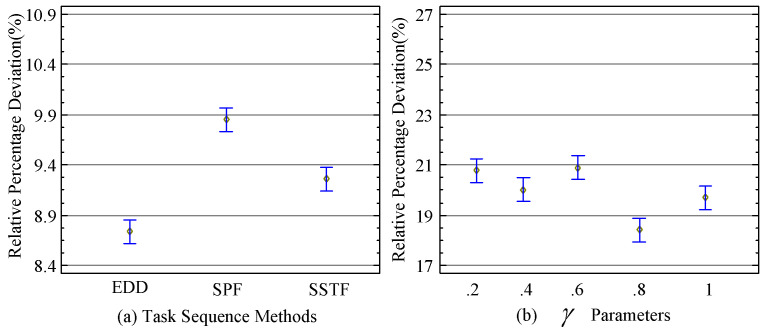
Task sequencing Tightness Rules With Different Rules.

Obtained results were based on delay that was calculated in microseconds (ms), as shown in Table 5. Execution delay (ms) and deadlines for all applications are shown in Table 5. LSEOS outperformed all existing methods in terms of delay and deadline, as shown in Figure 5.

### 5.3. Workflow Task Deadline Division

The workflow divided deadlines into tasks on the basis of their execution and communication time. This technique helps in how to execute all tasks under their deadlines onto different nodes. In the experiment, we divided the single workflow into task deadlines on the basis of Algorithm 2. All divisions were peformed before offloading and scheduling in the system.

### 5.4. Task Sequencing Rules of Workflow Tasks

The tigthness of values start from 0.2 not 0 because, in our model we show that 0.2 is the inital deadline and 0 means not scheduled in the distributed mobile edge cloud network. The task sequence component is important before task scheduling onto heterogeneous computing nodes. Different topological task sequencing rules are proposed that consist of EDD, SPD, and SSTF rules. Due to the different characteristics of tasks, the deadline, security, and availability of resources sorting all tasks with one rule is not enough. Therefore, we sorted all tasks into three sequence rules. All tasks were first sorted on the basis of EDD, and then they were sorting-based SPD. Lastly, on the basis of the best availability of resources, we sorted all tasks on the SSTF rule as shown in Figure 5a; EDD worked excellently as compared to others.

### 5.5. Assignment Delay Performance

These studies implemented as the baseline approaches that are closely related existing schemes in the simulation, for instance, delay optimal long short-term memory (LSTM) [21], workflow metaheuristic system (WFMS) [22], and workflow metaheuristic cloud (WMC) [23]. These studies are closely related to our work to execute the workflow applications on the heterogeneous nodes in mobile edge cloud computing.

### 5.6. Lightweight Secure Offloading

**Figure 6:**
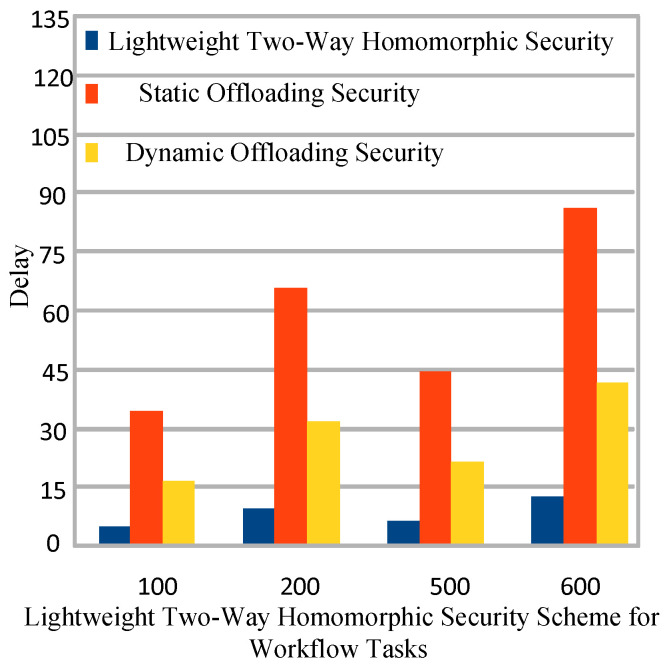
Lightweight Secure Offloading of Workflow Applications.

The lightweight mechanism shows the less delay execution during secure offloading between nodes in the study. The study devises the two secure lightweight homomorphic encryption secure scheme which only encrypt and decrypt data at the mobile devices instead of fog node and cloud. The main goal is to minimize the security delay at different level of nodes as existing static [11] and dynamic [1] offloading did in for the workflow applications. Figure 6 shows that, the proposed two ways lightweight homomorphic secure scheme outperformed all existing schemes for the mobile workflow applications in the system.

### 5.7. Adaptive Task Scheduling

**Figure 7:**
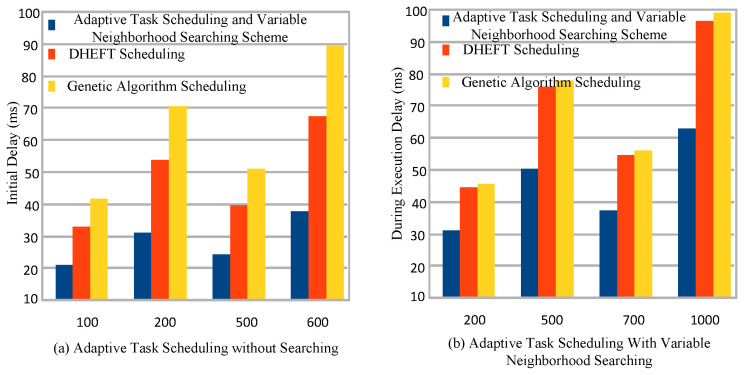
Adaptive task-scheduling and neighborhood-searching scheme for workflow applications.

The scheduling schemes DHEFT [1] and genetic algorithm [5] suggested the static and dynamic scheduling algorithms to run the workflow applications in mobile edge cloud network. The study devises the initial scheduling at different nodes gain the lower delay as shown in Figure 7a gain lower delay as compared to existing both DHEFT and genetic scheduling schemes. Figure 7b shows the adaptive delay optimal searching nodes based neighborhood searching enabled scheduling in the distributed mobile fog cloud network. The result shows that, the proposed adaptive searching technique search the delay optimal nodes at the run time of workflow applications in the system.

### 5.8. Delay Optimal Task Assignment

**Figure 8:**
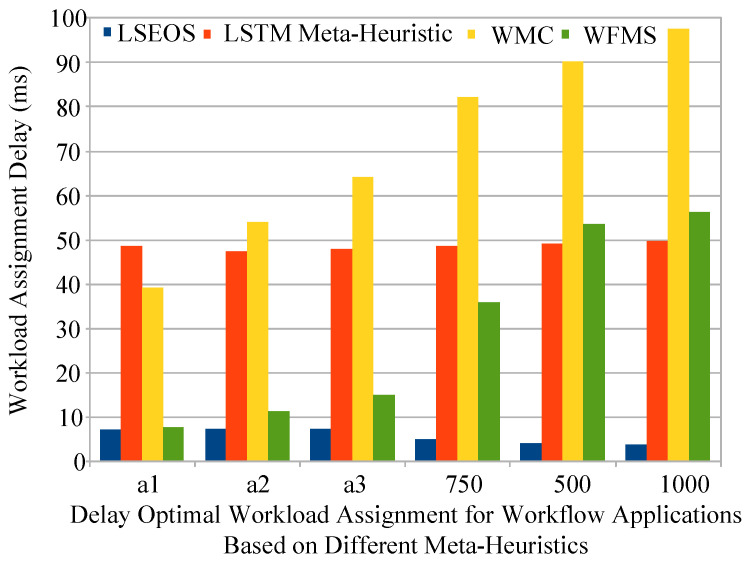
Lightweight workflow task assignment delay performance.

Figure 8 (a) analyzes the delay performance of all offloading and schemes on workflow applications in a heterogeneous mobile cloud environment. Proposed scheme LSEOS outperformed all existing schemes of LSTM, WMC, and WFMS metaheuristics with all workflow applications in terms of delay and workload assignment in mobile fog cloud networks. There are many reasons why these schemes have higher delay as compared to that of the proposed scheme. These studies suggested workflow schemes based on single node either on the mobile device, edge node or cloud computing. Due to the high complexity of workflows and their intermediate task dependency, the workflow application could not be at same place because their applications are so much heavier. For instance, mobile devices cannot run these applications alone at the local device due to resource-constraint issues. However, edge nodes can only run the workflow with minimal delay, as data cannot be stored on edge nodes due to their limited resources in the network. Therefore, cloud computing can store data, but due to the long distance, the cloud has longer execution and communication delay for workflow applications. The LSEOS divided applications into three different parts and achieved less delay due to the division of applications and executing them under their deadline.

### 5.9. Lightweight Secure Adaptive Offloading and Scheduling Approaches

This section outlines the achievements of methods that are lightweight secure adaptive offloading and scheduling approaches in the system.

**Figure 9:**
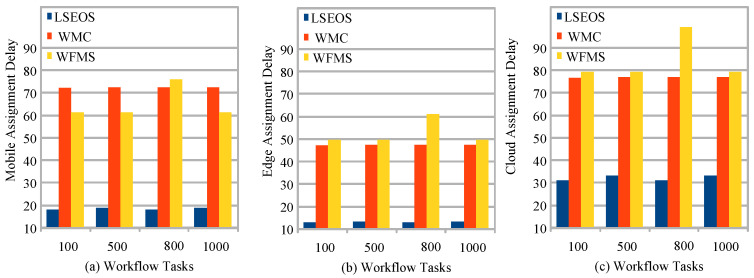
Lightweight secure adaptive offloading and scheduling workflow task performance: (**a**) Mobile Assignment Delay, (**b**) Edge Assignment Delay, (**c**) Cloud Assignment Delay.

Figure 9a–c show the lightweight workflow task assignment performance with the security validation and their deadline in the heterogeneous mobile edge cloud. The result shows that, the LSEOS outperformed all existing schemes because of the lightweight security scheme as compared existing secure offloading and scheduling in terms of delay for the healthcare workflow applications. LSEOS only encrypts and decrypts on local devices; therefore, it achieved a high security ratio, and edge and cloud nodes do not need to decrypt them. In this way, encryption and decryption time, and security validation can be widely improved for workflow applications when they are executed on different heterogeneous nodes.

### 5.10. Findings and Limitations

This paper devised the lightweight, secure offloading and task scheduling (LSEOS) algorithm framework, consisting of different schemes. The objective is to run workflow applications on other nodes and minimize the delay and security risk in the system. An adaptive deadline, sorting, and scheduling with neighborhood search schemes are suggested in the proposed model. This study found the following: (1) a lightweight security method was designed that minimized the security resources and time for the applications; (2) processing delays were minimized during offloading and scheduling in the system; (3) different sequencing rules were devised that satisfied the deadlines, priorities, and quality of service of applications. However, there are a few limitations to the proposed work. The study only supported the workflow application and did not support the coarse- and fine-grained workload in the system. Furthermore, the processing cost and security cost enabled constraints are very important during offloading and scheduling and were not considered in the system.

## 6. Conclusions and Future Work

This paper devised the lightweight secure offloading and task scheduling (LSEOS) algorithm framework, which consists of different schemes. The objective is to run workflow applications on other nodes and minimize the delay and security risk in the system. The adaptive deadline, sorting, and scheduling with neighborhood search schemes are suggested in the proposed model. The simulation results showed that the SEOS outperforms all existing offloading and scheduling methods for the workflow applications compared to current techniques for the delay and security validation in the model. There are many challenges exist in the current manuscript version that did not consider the block to block type security and validation in the proposed architecture. Mobility aspects of users were not investigated in the current architecture; therefore, future work will consider node to node validation and mobility of the users in the proposed architecture. 

## Figures and Tables

**Table 1:** Existing Internet of Medical Things approaches in IoT networks.

Studies	Approaches	Workload	Fog-Cloud Environment
[1,2]	Genetic Algorithm-SHA-256	Coarse-Grained	Homogeneous
[3,4]	HEFT-PSO-MD5-256	Fine-Grained	Homogeneous
[5,6]	Ant-Colony-CRC-256	Coarse-Grained	Homogeneous
[7,8]	Simulated-Brute-Force-256	Coarse-Grained	Heterogeneous
[9,10]	Genetic Algorithm-DES-256	Coarse-Grained	Heterogeneous
[11,12]	Genetic Algorithm-3DES-256	Coarse-Grained	Heterogeneous
[13,14]	Genetic Algorithm-SHA-256	Coarse-Grained	Heterogeneous
[15,16]	Genetic Algorithm-SHA-256	Coarse-Grained	Heterogeneous
[17,18]	Genetic Algorithm-SHA-256	Coarse-Grained	Homogeneous
[19,20]	Genetic Algorithm-SHA-256	Coarse-Grained	Heterogeneous
[21,22]	LSTM and WMC	Coarse-Grained	Homogeneous
[23,24]	WFMS	Coarse-Grained	Heterogeneous
[25,26,27,28,29,30]	KNN-DES-256	Coarse-Grained	Heterogeneous

**Table 2:** Problem notations.

Symbol	Purpose
*P*	Number of workflow applications
*A*	Particular workflow application
*mi*	Mobile tasks of workflow application a (local tasks)
*i*	Specific task of application a
*ei*	Edge tasks of workflow a (delay-sensitive tasks)
*ci*	Cloud computing tasks of workflow application a (delay-tolerant tasks)
$w_{a}$	Workload of workflow application a
$deadline_{a}$	Deadline of workflow a
*m*	Mobile node
*e*	Edge/fog node
*c*	Cloud node
$M_{e}^{mi}$	Execution time of a task at mobile device
$E_{e}^{ei}$	Execution time of a task at edge computing
$C_{e}^{ci}$	Execution time of a task at cloud computing
$\zeta_{m}$	Mobile computing node
$\epsilon_{m}$	Resource of mobile node
$\zeta_{k}$	Edge node speed
$\epsilon_{k}$	Resource of edge node
$\zeta_{c}$	Cloud computing node
$\epsilon_{c}$	Resource of cloud computing node
me	Communication time between mobile and edge nodes
ec	Communication between edge computing and cloud computing
$x_{mi=1}$	Assignment of tasks to mobile device
$x_{ei=2}$	Assignment of tasks to edge computing
$x_{ci=3}$	Assignment of tasks to cloud computing

**Table 3:** Lfogsim-based experimental parameters.

Simulation Parameters	Values
Setup	IFogsim
Workflow	Healthcare config file
Evaluation Methods	Statistical median
Mobile Node	HTC G17 and Samsung 1997
Edge Node	Intel 5 laptop, AndroidX86
Public Node	AndroidX86 Amazon t2.medium
me	10–30 ms
ec	100 ms
Performance criteria	Security and delay, deadline, and data validation based on Equations (6) and (9).

**Table 4:** Workflow tasks.

Workflow Application	Mobile Tasks	Edge Tasks	Cloud Tasks	File
a1	100	500	1000	Configuration
a2	50	400	700	Configuration
a3	90	600	800	Configuration

**Table 5:** Delay optimal workload assignment.

Application	Methods	Delay (ms)	Deadline (ms)
a1	LSEOS	1000	899
a1	LSTM Metaheuristic	1300	899
a1	WMC	1500	899
a1	WFMS	1000	899
a2	LSEOS	700	1000
a2	LSTM Metaheuristic	850	1000
a2	WMC	1200	1000
a2	WFMS	1100	1000
a3	LSEOS	320	500
a3	LSTM Metaheuristic	450	500
a3	WMC	700	500
a3	WFMS	670	500

## Data Availability

All the experimental data are generated at the local institution servers. Therefore, it cannot be made publicly available for other researchers.
